# Aged Mice Devoid of the M_3_ Muscarinic Acetylcholine Receptor Develop Mild Dry Eye Disease

**DOI:** 10.3390/ijms22116133

**Published:** 2021-06-07

**Authors:** Aytan Musayeva, Subao Jiang, Yue Ruan, Jenia Kouchek Zadeh, Panagiotis Chronopoulos, Norbert Pfeiffer, Werner E.G. Müller, Maximilian Ackermann, Ning Xia, Huige Li, Adrian Gericke

**Affiliations:** 1 Department of Ophthalmology, University Medical Center, Johannes Gutenberg University Mainz, Langenbeckstrasse 1, 55131 Mainz, Germany; sjiang@uni-mainz.de (S.J.); yruan@uni-mainz.de (Y.R.); je.kouchek@hotmail.de (J.K.Z.); panagiotis.chronopoulos@hotmail.com (P.C.); norbert.pfeiffer@unimedizin-mainz.de (N.P.); 2 Laboratory of Corneal Immunology, Transplantation and Regeneration, Schepens Eye Research Institute, Massachusetts Eye and Ear, Department of Ophthalmology, Harvard Medical School, Boston, MA 02114, USA; 3 ERC Advanced Investigator Grant Research Group at the Institute for Physiological Chemistry, University Medical Center, Johannes Gutenberg University Mainz, Duesbergweg 6, 55128 Mainz, Germany; wmueller@uni-mainz.de; 4 Institute of Functional and Clinical Anatomy, University Medical Center, Johannes Gutenberg University Mainz, Johann-Joachim-Becher-Weg 13, 55128 Mainz, Germany; maximilian.ackermann@uni-mainz.de; 5 Department of Pharmacology, University Medical Center, Johannes Gutenberg University Mainz, Langenbeckstrasse 1, 55131 Mainz, Germany; xianing@uni-mainz.de (N.X.); huigeli@uni-mainz.de (H.L.)

**Keywords:** cornea, dry eye disease, M_3_, muscarinic receptor, tear secretion

## Abstract

The parasympathetic nervous system is critically involved in the regulation of tear secretion by activating muscarinic acetylcholine receptors. Hence, various animal models targeting parasympathetic signaling have been developed to induce dry eye disease (DED). However, the muscarinic receptor subtype (M_1_–M_5_) mediating tear secretion remains to be determined. This study was conducted to test the hypothesis that the M_3_ receptor subtype regulates tear secretion and to evaluate the ocular surface phenotype of mice with targeted disruption of the M_3_ receptor (M3R^−/−^). The experimental techniques included quantification of tear production, fluorescein staining of the ocular surface, environmental scanning electron microscopy, assessment of proliferating cells in the corneal epithelium and of goblet cells in the conjunctiva, quantification of mRNA for inflammatory cytokines and prooxidant redox enzymes and quantification of reactive oxygen species. Tear volume was reduced in M3R^−/−^ mice compared to age-matched controls at the age of 3 months and 15 months, respectively. This was associated with mild corneal epitheliopathy in the 15-month-old but not in the 3-month-old M3R^−/−^ mice. M3R^−/−^ mice at the age of 15 months also displayed changes in corneal epithelial cell texture, reduced conjunctival goblet cell density, oxidative stress and elevated mRNA expression levels for inflammatory cytokines and prooxidant redox enzymes. The findings suggest that the M_3_ receptor plays a pivotal role in tear production and its absence leads to ocular surface changes typical for DED at advanced age.

## 1. Introduction

Dry eye disease (DED) is a common chronic condition that occurs when tears do not provide adequate lubrication for the ocular surface, which is characterized by tear film instability, increased tear osmolarity and ocular surface inflammation [1]. DED may cause pain and ocular surface epitheliopathy with consecutive loss of quality of life and vision [1,2]. DED can be classified into aqueous deficient disease due to lacrimal hyposecretion, and into aqueous sufficient disease due to Meibomian gland disease or altered tear spread [1]. Previous studies have shown that aqueous deficiency causes dysfunction and loss of conjunctival goblet cells, while goblet cell number has been reported to remain in the normal range in Meibomian gland disease [3]. There are numerous causes for aqueous deficiency, such as aging, injury, anticholinergic medication, systemic inflammatory/immune diseases, e.g., Sjögren’s syndrome, as well as diseases that affect neural innervation or signaling [1,4,5,6]. To study the underlying disease mechanisms, an increasing number of models for DED are being established reflecting the growing interest in the pathophysiology of the disease [7,8,9,10]. The parasympathetic nervous system is involved in tear secretion via the activation of muscarinic acetylcholine receptors in the lacrimal gland [11]. Hence, various pharmacological techniques aimed at targeting parasympathetic pathways have been developed to study DED in animal models. Most of these models are based on either transdermal application, subcutaneous injection or implantation of devices enabling a continuous delivery of muscarinic receptor blockers, such as scopolamine or atropine [7,11,12]. The applied antagonists block more than one of the five muscarinic receptor subtypes (M_1_–M_5_) resulting in various side-effects. The lack of highly selective pharmacological ligands and antibodies for individual muscarinic receptor subtypes may be the reason why the muscarinic receptor subtype mediating tear secretion has not been clearly determined so far [13,14,15]. Based on studies reporting that some patients with primary Sjögren’s syndrome carry inhibitory autoantibodies against the M_3_ receptor subtype, this receptor was suggested to be involved in mediating tear secretion [16,17]. However, this evidence is only indirect. The goal of the present study was to examine the hypothesis that the M_3_ muscarinic acetylcholine receptor subtype is involved in regulation of tear secretion. For our studies, we used mice with targeted disruption of the M_3_ receptor (M3R^−/−^) to circumvent the problem of limited subtype selectivity of pharmacological ligands. Another objective of this study was to test whether M_3_ receptor deficiency is associated with ocular surface alterations typical for DED due to aqueous deficiency.

## 2. Results

### 2.1. Tear Production and Ocular Surface Characteristics

**Figure 1:**
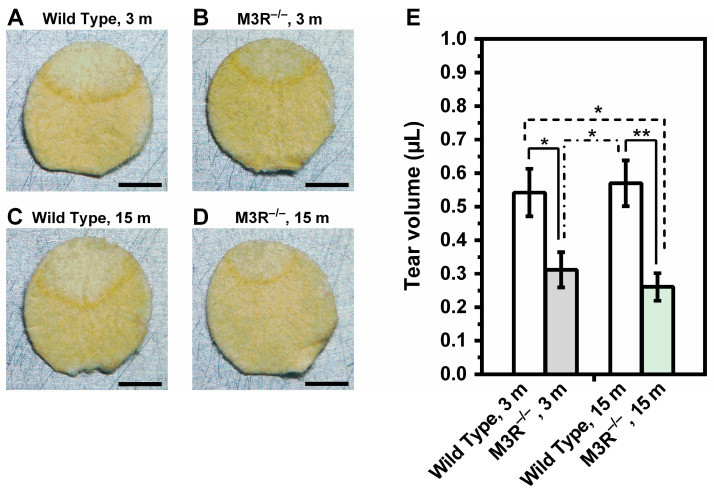
Determination of tear production in the wild-type and M3R^−/−^ mice by using a round piece of absorbent paper. Tear production was reduced in M3R^−/−^ mice at the age of 3 months (m) compared to wild-type mice of the same age (**A**,**B**,**E**). At the age of 15 m, the difference in tear production between M3R^−/−^ and wild-type mice was slightly more pronounced (**C**–**E**) (* *p* < 0.05, ** *p* < 0.01, *n* = 8 per genotype and age category). Scale bar = 2 mm.

Tear volume was markedly reduced in the M3R^−/−^ mice compared to the wild-type mice at the age of 3 months (0.3116 ± 0.05248 µL vs. 0.5420 ± 0.07092 µL; M3R^−/−^ vs. wild-type at 3 months). At the age of 15 months, no significant age-dependent changes in tear fluid volume were observed in either genotype. However, the difference in tear volume between the M3R^−/−^ vs. the wild-type was slightly more pronounced at the age of 15 months (0.2609 ± 0.04102 µL vs. 0.5697 ± 0.06821 µL; M3R^−/−^ vs. wild-type at 15 months) compared to the 3-month-old mice (*n* = 8 per genotype and age category, * *p* < 0.05, ** *p* < 0.01, Figure 1).

**Figure 2:**
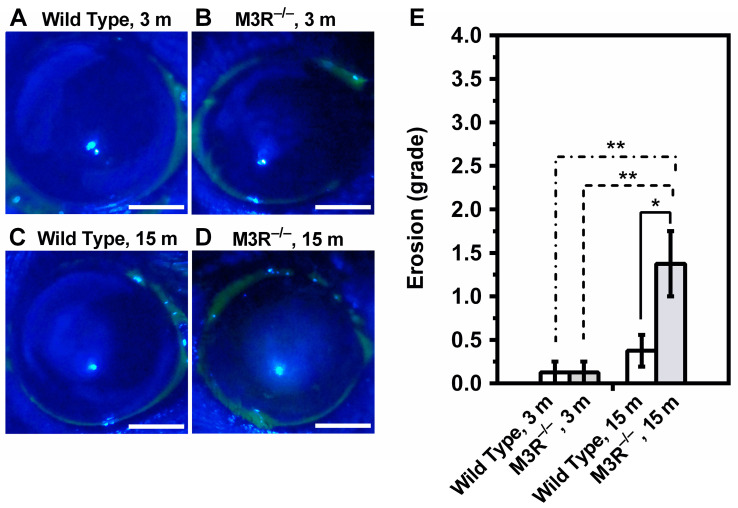
Determination of ocular surface epitheliopathy by fluorescein staining in the 3-month-old wild-type (**A**) and M3R^−/−^ mice (**B**) and in the 15-month-old wild-type (**C**) and M3R^−/−^ mice (**D**). Epitheliopathy was assessed based on the Oxford Scheme grading system. M3R^−/−^ mice at the age of 15 months had mild but significant epitheliopathy (**E**) (* *p* < 0.05, ** *p* < 0.01, *n* = 8 per genotype and age category). Scale bar = 1 mm.

Exposure of the ocular surface to fluorescein revealed virtually no stained spots in the 3-month-old M3R^−/−^ (Figure 2A) and wild-type mice (Figure 2B). In contrast, the 15-month-old M3R^−/−^ mice had markedly more stained spots in the corneal surface compared to the 15-month-old wild-type mice and compared to the 3-month-old M3R^−/−^ and wild-type mice (Figure 2C–E). According to the Oxford Scheme grading system, this corresponded to mild keratopathy (*n* = 8 per genotype and age category, * *p* < 0.05, ** *p* < 0.01).

**Figure 3:**
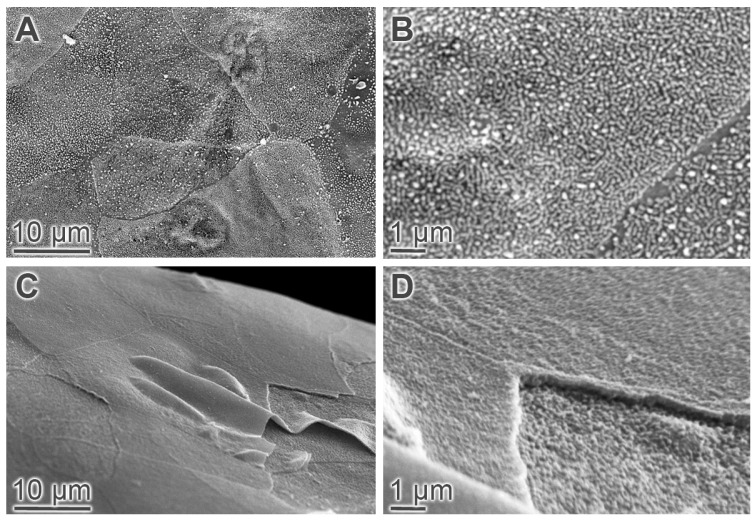
ESEM analysis of the corneal epithelium. The corneal epithelium of the 15-month-old wild-type mice (**A**) was composed of polygonal cells with a shaggy texture, a high density and length of microvilli and well-defined cell borders (**B**). In the 15-month-old M3R^−/−^ mice, the texture of the cellular surface appeared smooth. Delaminating superficial epithelial cells were frequently seen (**C**). Microvilli were much shorter in the 15-month-old M3R^−/−^ mice compared to the age-matched wild-type mice (**D**).

An ESEM analysis of the wild-type mouse cornea at the age of 15 months revealed a relatively uniform surface appearance with polygonal cells of a shaggy texture and a high density of long microvilli. Moreover, the borders between most cells were well-defined, indicative of substantial cell to cell contact (Figure 3A,B). In the M3R^−/−^ mouse cornea at the age of 15 months, the epithelial cell surface appeared smooth due to fewer and shorter microvilli, and the borders between cells were less defined. Moreover, delaminating superficial epithelial cells were frequently visible in mutant mice (Figure 3C,D).

### 2.2. Corneal Epithelial Cell Proliferation and Conjunctival Goblet Cell Density

**Figure 4:**
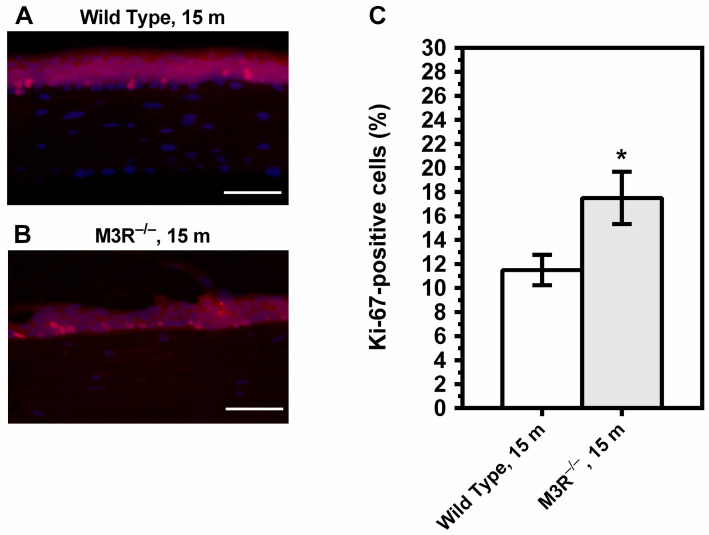
Cross-sections of the corneal epithelium from the 15-month (m)-old wild-type (**A**) and M3R^−/−^ mice (**B**) stained with an antibody directed against the proliferation marker Ki-67 (red) and with DAPI (blue). The M3R^−/−^ mice had a markedly higher rate of proliferating cells in the epithelium (**C**). (* *p* < 0.05, *n* = 8 per genotype). Scale bar = 100 µm.

The percentage of Ki-67-positive cells was increased in the corneal epithelium of the 15-month-old M3R^−/−^ mice (17.50 ± 2.179%) compared to the age-matched wild-type mice (11.50 ± 1.268%) (* *p* < 0.05, Figure 4). Ki-67-positive cells were not seen in the corneal stroma or endothelium.

**Figure 5:**
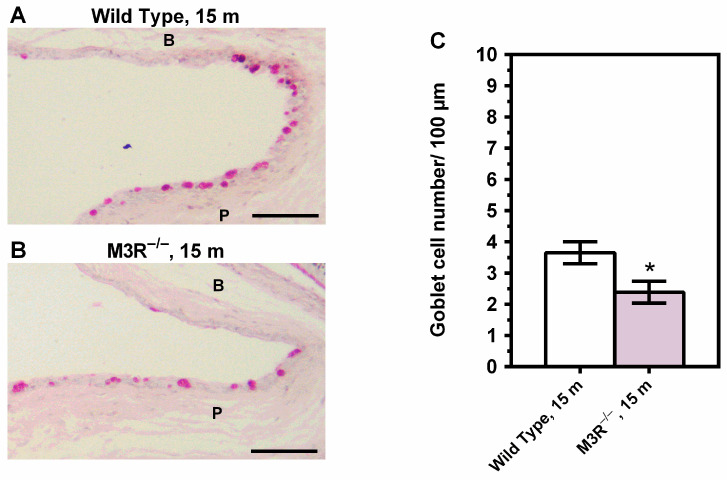
PAS-stained cross-sections of the conjunctival epithelium from the wild-type (**A**) and M3R^−/−^ mice (**B**) at the age of 15 months (m). The M3R^−/−^ mice had a markedly reduced density of goblet cells in the palpebral conjunctiva (**C**) (* *p* < 0.05, *n* = 8 per genotype). B = bulbar conjunctiva; P = palpebral conjunctiva; Scale bar = 100 µm.

The density of goblet cells in the goblet-cell-rich palpebral conjunctiva was markedly lower in the 15-month-old M3R^−/−^ mice (2.388 ± 0.3507 cells/100 µm) compared to the wild-type mice (3.650 ± 0.3541 cells/100 µm) (*n* = 8 per genotype, * *p* < 0.05, Figure 5).

### 2.3. Expression of Inflammatory and Prooxidant Redox Genes

**Figure 6:**
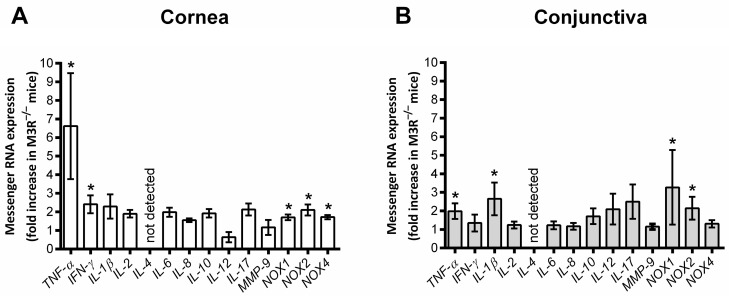
Messenger RNA expression for inflammatory cytokines and prooxidant redox enzymes in the corneal (**A**) and conjunctival epithelium (**B**) of the 15-month-old mice. Data are presented as the fold-change (mean ± SE) in M3R^−/−^ versus wild-type mice (*n* = 8 per genotype, * *p* < 0.05).

In the corneal epithelium of the 15-month-old M3R^−/−^ mice, the mRNA expression for the inflammatory cytokines TNF-α and IFN-γ was increased compared to the age-matched wild-type mouse epithelium. In addition, mRNA levels for the prooxidant redox enzymes NOX1, NOX2 and NOX4 were markedly elevated. In the conjunctival epithelium of the M3R^−/−^ mice, TNF-α mRNA expression was also increased. Moreover, the mRNA levels for IL-1β and for NOX1 and NOX2 were higher compared to the wild-type mice (*n* = 8 per genotype, Figure 6).

### 2.4. ROS Formation in the Corneal and Conjunctival Epithelium

**Figure 7:**
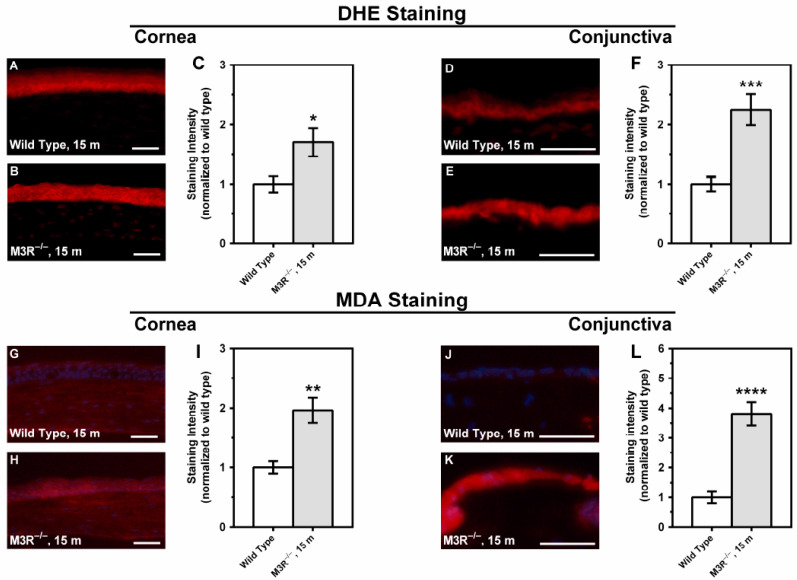
DHE stainings of corneal cross-sections from the 15-month-old wild-type (**A**) and age-matched M3R^−/−^ mice (**B**). Staining intensity was increased in the corneal epithelium from the M3R^−/−^ mice (**C**). DHE stainings were also conducted in the conjunctiva from the 15-month-old wild-type (**D**) and age-matched M3R^−/−^ mice (**E**). The intensity of the DHE fluorescence was also markedly increased in the conjunctival epithelium from the M3R^−/−^ mice (**F**). Moreover, immunostainings were performed in corneal cross-sections from the 15-month-old wild-type (**G**) and M3R^−/−^ mice (**H**) using an antibody directed against MDA. The staining intensity for MDA was also increased in the corneal epithelium of the M3R^−/−^ mice (**I**). MDA stainings in conjunctival cross-sections from the wild-type (**J**) and M3R^−/−^ mice (**K**) revealed much more intense staining in the M3R^−/−^ mice (**L**). Values are presented as mean ± SE (* *p* < 0.05, ** *p* < 0.01, *** *p* < 0.001, **** *p* < 0.0001, *n* = 8 per genotype). Scale bar = 50 µm.

DHE and MDA staining revealed increased fluorescence intensity in both corneal and conjunctival epithelium of the 15-month-old M3R^−/−^ mice compared to the age-matched wild-type mice, which is indicative of elevated ROS levels (*n* = 8 per genotype; Figure 7). The differences in fluorescence intensity between the M3R^−/−^ and wild-type mice were more pronounced in the conjunctival epithelium.

## 3. Discussion

There are several major new findings emerging from the present study. First, tear production was markedly reduced in the M3R^−/−^ mice already at the age of three months, suggesting that the M_3_ receptor participates in regulation of tear secretion. Second, the 15-month-old, but not the 3-month-old, M3R^−/−^ mice developed ocular surface epitheliopathy. These changes were mild but had characteristics of DED, such as loss of microvilli and epithelial cell desquamation and were associated with an increased proliferation rate of corneal epithelial cells and a reduced amount of goblet cells in the conjunctival epithelium. Third, the 15-month-old M3R^−/−^ mice displayed elevated mRNA levels for inflammatory cytokines and prooxidant redox genes in both the corneal and conjunctival epithelium. Likewise, the ROS levels were elevated in corneal and conjunctival epithelium of the M3R^−/−^ mice at the age of 15 months. Altogether, these findings indicate that the M3R^−/−^ mice are characterized by tear deficiency at young age already but develop morphological changes associated with DED at advanced age.

Because parasympathetic nerves contribute to tear secretion from the lacrimal gland, several animal models have been established for studies of DED that are based on pharmacological, mechanical or genetic inactivation of parasympathetic signaling [11,18]. Most of these animal models are either invasive or require repeated administration of drugs, which makes the models rather challenging especially for longitudinal studies of DED. In the established animal models, drugs have been used that block all five muscarinic receptor subtypes, hence, their systemic administration may induce side-effects in various organs [19]. The use of recently developed selective ligands for individual muscarinic receptors may reduce the systemic side effects [14,20], but is not likely to reduce the invasiveness and challenges of repeated drug administration. The use of genetically modified mice lacking individual muscarinic receptor subtypes may circumvent these problems. The findings of the present study suggest that a major portion of the parasympathetic input to the lacrimal gland is mediated by the M_3_ receptor. Since the M3R^−/−^ mice are viable, fertile and have a normal life expectancy, they may constitute an attractive model for longitudinal studies of DED [21,22]. Notably, tear secretion did not markedly change in the M3R^−/−^ and wild-type mice with advancing age. Previous studies reported either an increase or no change in C57BL/6 mouse tear volume with advancing age [23,24].

Our findings also demonstrate that the M3R^−/−^ mice develop structural changes in the corneal epithelium, such as epithelial defects and a reduction of superficial corneal epithelial microvilli with advancing age. Since microvilli of the superficial epithelium are important for tear film anchorage, their reduction may result in tear film instability. Moreover, the rate of proliferating cells was increased in the corneal epithelium of the 15-month-old M3R^−/−^ mice, a typical observation in DED, which may be regarded as a compensatory reaction against dry eye-induced epithelial damage [25,26].

We also found that the number of goblet cells was reduced in the conjunctival epithelium from 15-month-old M3R^−/−^ mice. Previous studies in humans reported that aqueous deficiency causes dysfunction and loss of conjunctival goblet cells, while goblet cell number has been reported to remain within the normal range in Meibomian gland disease [3]. Likewise, desiccating stress was shown to induce conjunctival goblet cell loss in animal models [27,28], which is in part mediated by IFN-γ and CD4+ T cells [29,30,31]. Messenger RNA levels for some pro-inflammatory cytokines, such as IFN-γ, IL-1β and TNF-α, were increased in the corneal and/or conjunctival epithelium of the 15-month-old M3R^−/−^ mice, indicative of ocular surface inflammation. However, other cytokines usually involved in DED, such as IL-6 or IL-8, were not elevated, which may suggest that ocular surface inflammation was only mild. Cytokines, such as TNF-α and IL-1β, were shown to activate matrix metalloproteinase 9 (MMP-9) expression and activity on the ocular surface in DED [32]. 

MMP-9 is a proteolytic enzyme that acts on extracellular matrix and cell surface adhesion molecules [33]. An increased concentration and activity of MMP-9 has been observed in the tear fluid of dry eye patients and experimental DED animal models [34,35,36,37,38]. Potential sources of MMP-9 include immune cells in the ocular surface and activated corneal and conjunctival epithelial cells [39,40]. Studies in genetically modified mice revealed that increased MMP-9 activity substantially contributes to the disruption of the corneal epithelial barrier in DED [39]. In previously reported mouse models of DED created by administration of anticholinergic agents, the mRNA levels for MMP-9 were markedly increased in the corneal and/or conjunctival epithelium [36,40]. Notably, the MMP-9 mRNA expression was not elevated in the cornea and conjunctiva of the M3R^−/−^ mice. This finding may further support the notion that DED was only mild in the M3R^−/−^ mice because MMP-9 activity, mRNA and protein levels were shown to correlate with the severity of DED [38,41]. However, since we did not determine MMP-9 concentration and activity in the tear fluid, we cannot rule out the possibility MMP-9 concentration and/or activity was increased in the M3R^−/−^ mice. Previous studies in human corneal epithelial cells (HCECs) and in in vivo DED models suggested that oxidative stress is involved in the pathogenesis of DED [42,43,44,45]. We found that the levels of reactive oxygen species (ROS) were elevated in the corneal and conjunctival epithelium. Moreover, we found that mRNA expression for the prooxidant nicotinamide adenine dinucleotide phosphate oxidase (NOX) isoforms NOX1, NOX2 and NOX4 was elevated in the corneal epithelium and for the isoforms, NOX1 and NOX2, in the conjunctival epithelium, suggesting that NOX may contribute to oxidative stress in the ocular surface epithelium in DED. The role of individual NOX isoforms in the pathogenesis of DED should be pursued further, since they may constitute potential therapeutic targets.

In summary, the lack of the M_3_ muscarinic acetylcholine receptor results in reduced tear secretion already at young age, but corneal epitheliopathy develops with advancing age. The changes of the ocular surface observed in the aged M3R^−/−^ mice were typical for DED. However, the observed corneal epitheliopathy was only mild in the aged M3R^−/−^ mice, suggesting that the morphological changes due to reduced tear secretion were relatively subtle. The fact that mRNA for relatively few inflammatory cytokines was elevated in the corneal and conjunctival epithelium also supports the notion that DED was relatively mild in the M3R^−/−^ mice. Mild corneal changes have also been reported in mice subjected to lacrimal gland excision [7], suggesting that the residual tear supply was sufficient to prevent pronounced signs of ocular surface disease. Therefore, such models are often combined with environmental stress factors, such as desiccation by using an air blower [7]. Based on our findings, the M3R^−/−^ mice appear to be an attractive, non-invasive alternative to existing dry eye models, which would facilitate longitudinal studies of DED. From a clinical point of view, selective activation of the M_3_ receptor might become therapeutically useful to promote tear secretion with less side effects compared to non-subtype-selective muscarinic receptor ligands.

## 4. Materials and Methods

### 4.1. Animals 

The study was approved by the Animal Care Committee of Rhineland-Palatinate, Germany (approval number: AZ 23 177-07/G 13-1-057), and all animals were treated in accordance with the EU Directive 2010/63/EU for animal experiments. The M_3_ receptor knockout mice (M3R^−/−^) and respective wild-type controls of the C57BL/6NTac background were used for the study. The generation of M3R^−/−^ mice has been described previously [21]. The M3R^−/−^ mice were backcrossed with C57BL/6NTac mice for 10 generations to obtain N10 congenic mice. Polymerase chain reaction (PCR) of DNA isolated from tail biopsies was used to identify the genotype of each animal. The M3R^−/−^ mice are complete knockouts with no remaining M_3_ receptor activity. The mice were housed under pathogen-free conditions with a 12 h light/dark cycle, a temperature of 22 ± 2 °C, a humidity of 55 ± 10% and with free access to food and tap water. In all experiments, male mice at the ages of 3 and 15 months were used. The calculated number of animals per group was 8 assuming an effect size of d = 1.8 for tear secretion (power = 90%, α = 0.05).

### 4.2. Quantification of Tear Production

**Figure 8:**
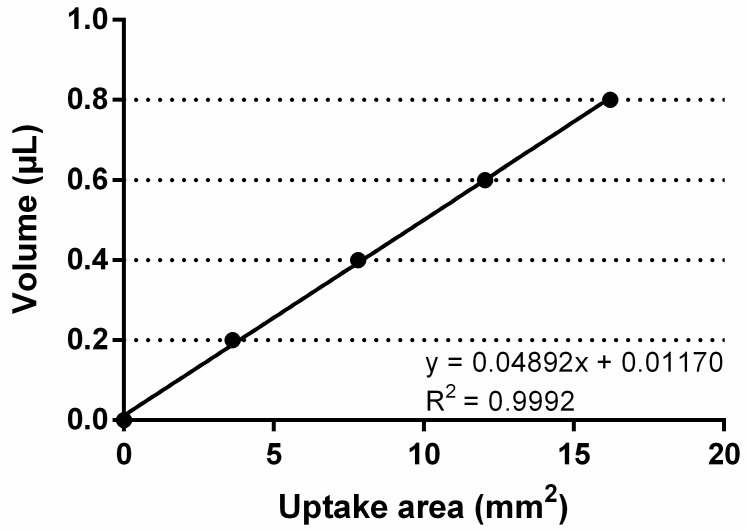
Standard curve of fluid uptake using absorbent paper and known volumes of a basic solution (0.9% NaCl). The resulting uptake areas were plotted against volume. This curve was then used to convert actual mouse tear uptake areas into tear volume.

After animals had been anesthetized by ketamine/xylazine, tear production was determined by using a round piece of absorbent paper of 6 mm diameter, which was folded in the middle, taken with a pair of tweezers and immersed into the tear meniscus on the lower conjunctival fornix for 20 sec (left and right eye). Next, the paper was photographed under a microscope and the moistened area determined with Image J (NIH, http://rsb.info.nih.gov/ij/ (accessed on 11 March 2019)). The measured uptake of tear fluid in mm^2^ was compared to a standard curve prepared using absorbent paper to uptake known volumes of a stock basic solution (0.9% NaCl) (Figure 8).

### 4.3. Characterization of the Corneal Surface

To assess the occurrence of ocular surface epitheliopathy, the eyes of anesthetized mice (ketamine/xylazine) were stained by placing 2 µL of 1% sodium fluorescein onto the ocular surface. After 3 min, photographs were taken under cobalt blue light using a stereomicroscope. Punctate staining was evaluated by a blinded evaluator (J.K.Z.) using the Oxford Scheme grading system, and a grade of 0 to 4 was assigned as described previously [46,47].

### 4.4. Environmental Scanning Electron Microscopy

To characterize corneal epithelial cell structure in more detail, we conducted studies by environmental scanning electron microscopy (ESEM, ESEM XL-30, Philips, Eindhoven, The Netherlands) as previously reported [48]. For this analysis, isolated corneas were transferred into 2% (*v*/*v*) aqueous glutaraldehyde fixative, fixated in osmium oxide, processed through acetone dehydration steps and finally critical point dried at 43 °C.

### 4.5. Quantification of Proliferating Corneal Epithelial Cells and Conjunctival Goblet Cells

The whole eyeball together with the eyelids and conjunctiva was embedded in Tissue-Tek^®^ O.C.T.™ compound (Sakura Finetek Germany GmbH, Staufen, Germany) and snap frozen in liquid nitrogen. Actively proliferating cells in the corneal epithelium were identified by immunofluorescence on central sagittal eye globe cryosections (10 μm) using an antibody directed against the Ki-67 protein, a cell proliferation marker expressed during active phases of the cell cycle [49]. Tissue sections were fixed in 4% paraformaldehyde for 20 min and washed in phosphate-buffered saline (PBS) twice for 5 min. Subsequently, the tissue sections were blocked with 1% bovine serum albumin for 30 min. The primary antibody (Ki-67 monoclonal antibody from rabbit, MA5-14520, Thermo Fisher Scientific GmbH, Dreieich, Germany) was diluted (1:300 in PBS), placed on the sections and incubated overnight at 4 °C. Next, the slices were washed three times in PBS for 5 min and the secondary antibody (goat anti-rabbit IgG H&L, TRITC-conjugated, catalog number: ab6718, Abcam, Cambridge, UK), diluted 1:200 in PBS, was applied for 1 h in darkness. Subsequently, slides were washed in PBS again and were mounted using a mounting medium containing 4′6-diamidino-2-phenylindole (DAPI) (Vectashield Mounting Medium with DAPI, catalog number: H-1200, BIOZOL Diagnostica Vertrieb GmbH, Eching, Germany) to stain the cell nuclei. Tissue sections were viewed and photographed under a fluorescent microscope (Nikon Eclipse E800) equipped with a digital SPOT camera (Nikon Inc., Tokyo, Japan). DAPI- and Ki-67-labelled cells were counted through the entire thickness of the corneal epithelium in a central zone of 880 µm width by a masked evaluator (A.M.) as previously described [50]. The rate of proliferating cells was defined as the percentage of Ki-67-positive cells of all DAPI-positive cells. To determine the number of goblet cells in the conjunctiva, cryosections of 10 µm thickness were cut and stained with periodic acid-Schiff (PAS) reagent. Conjunctival goblet cell density was measured by a blinded evaluator (S.J.) over 100 µm lengths on the palpebral conjunctiva and results were expressed as the number of positive cells per 100 μm.

### 4.6. Quantification of Inflammatory and Prooxidant Redox Genes by Real-Time PCR

Messenger RNA levels for the inflammatory cytokines, TNF-α, INF-γ, IL-1β, IL-2, IL-4, IL-6, IL-8, IL-10, IL-12, IL-17 and MMP-9, and for the prooxidant redox enzymes, NOX1, NOX2 and NOX4, were determined in corneal and conjunctival epithelium from the 15-month-old wild-type and the M3R^−/−^ mice by real-time PCR. After the euthanasia of mice by CO_2_ exposure, one eye per mouse was immediately excised and transferred into cooled PBS (Invitrogen, Karlsruhe, Germany) to excise the cornea and conjunctiva under a dissecting microscope. The corneal epithelium was scraped off from the stroma by using fine-point tweezers to obtain corneal epithelial cells. After excision of the conjunctiva, the subepithelial conjunctival tissue was thoroughly removed by Vannas scissors. The isolated epithelia were transferred into 1.5 mL plastic tubes, rapidly frozen in liquid nitrogen and stored at −80 °C. Later, tissue samples were homogenized (FastPrep; MP Biomedicals, Illkirch, France), and the expression of genes was measured by SYBR Green-based quantitative real-time PCR, as previously described [51]. RNA was isolated using peqGOLD TriFast™ (PEQLAB) and cDNA was generated with the High Capacity cDNA Reverse Transcription Kit (Applied Biosystems, Darmstadt, Germany). Real-time PCR reactions were performed on a StepOnePlus™ Real-Time PCR System (Applied Biosystems) using SYBR^®^ Green JumpStart™ Taq ReadyMix™ (Sigma-Aldrich, Steinheim, Germany) and 20 ng cDNA. The relative mRNA levels of target genes were quantified using comparative threshold (CT) normalized to the TATA-binding protein (TBP) housekeeping gene. Messenger RNA expression is presented as the fold-change in the M3R^−/−^ mice versus the wild-type mice. The PCR primer sequences are listed in Table 1.

### 4.7. Quantification of Reactive Oxygen Species

ROS formation was determined in 10 µm cryosections of eye globes by dihydroethidium (DHE, 1 µM)-derived fluorescence. In the corneal and conjunctival epithelium, the fluorescence (518 nm/605 nm excitation/emission) was measured as previously described [52]. Moreover, immunostainings for malondialdehyde (MDA), a highly reactive three carbon dialdehyde, which is a marker for oxidative stress, have been performed. Briefly, frozen sections of 10 μm thickness were cut and fixed in 4% paraformaldehyde solution for 20 min. Next, slides were rinsed with PBS and incubated at room temperature with blocking solution containing 1% bovine serum albumin for 30 min. Next, the primary antibody directed against MDA (ALX-210-879, rabbit, polyclonal, Enzo Life Sciences, Inc., Farmingdale, NY, U.S.) was diluted (1:500) in blocking solution and incubated for 2 h at room temperature. Thereafter, each slide was washed in PBS three times for 5 min and incubated for 1 h at room temperature with a Rhodamine Red-X-coupled secondary antibody (111-295-003, goat anti-rabbit, polyclonal, dilution: 1:200, Dianova GmbH, Hamburg, Germany). Negative control sections were incubated with a blocking medium and the secondary antibody. Finally, slides were washed in PBS (3 × 5 min) and were mounted by using VECTASHIELD^®^ Mounting Medium with DAPI (BIOZOL Diagnostica Vertrieb GmbH, Eching, Germany) and cover-slipped. Subsequently, the fluorescence was measured in the corneal and conjunctival epithelium by using Image J (NIH, http://rsb.info.nih.gov/ij/ (accessed on 11 March 2019).

### 4.8. Statistics

Data are presented as mean ± SE, and n represents the number of mice per group. Normality was tested by the D’Agostino & Pearson omnibus normality test. Since values were normally distributed, one-way ANOVA with the Tukey’s multiple comparisons test (comparison of more than two groups) or an unpaired *t*-test (comparison of two groups) was used. The level of significance was set at 0.05.

## Figures and Tables

**Table 1:** Primer sequences used for quantitative PCR analysis.

Gene	Forward	Reverse
*TNF-α*	GCC TCT TCT CAT TCC TGC TTG	CTG ATG AGA GGG AGG CCA TT
*IFN-γ*	AGC GGC TGA CTG AAC TCA GAT TGT AG	GTC ACA GTT TTC AGC TGT ATA GGG
*IL-1β*	AAG GAG AAC CAA GCA ACG ACA AAA	TGG GGA ACT CTG CAG ACT CAA ACT
*IL-2*	CAA GTC CTG CAG GCA TGT ACA	CTG TTG ACA AGG AGC ACA AGT GT
*IL-4*	CGCCATGCACGGAGATG	CGAGCTCACTCTCTGTGGTGTT
*IL-6*	ACA ACC ACG GCC TTC CCT ACT T	CAC GAT TTC CCA GAG AAC ATG TG
*IL-8*	ACGGACATGGCTGCTCAAG	GGACGAAGATGCCTAGGTTAAGG
*IL-10*	CAT GGG TCT TGG GAA GAG AA	AAC TGG CCA CAG TTT TCA GG
*IL-12*	CAT CCA GCA GCT CCT CTC AGT	GCA AGG GTG GCC AAA AAG A
*IL-17*	CCT CAC ACG AGG CAC AAG TG	TCT CCC TGG ACT CAT GTT TGC
*MMP-9*	AAAGACCTGAAAACCTCCAACCT	GCCCGGGTGTAACCATAGC
*NOX1*	GGAGGAATTAGGCAAAATGGATT	GCTGCATGACCAGCAATGTT
*NOX2*	CCAACTGGGATAACGAGTTCA	GAGAGTTTCAGCCAAGGCTTC
*NOX4*	TGTAACAGAGGGAAAACAGTTGGA	GTTCCGGTTACTCAAACTATGAAGAGT
*TBP*	CTT CGT GCA AGA AAT GCT GAA T	CAG TTG TCC GTG GCT CTC TTA TT

## Data Availability

The data presented in this study are available on request from the corresponding author.
